# 3D analysis of the whole subcutaneous adipose tissue reveals a complex spatial network of interconnected lobules with heterogeneous browning ability

**DOI:** 10.1038/s41598-019-43130-9

**Published:** 2019-04-30

**Authors:** Jules Dichamp, Corinne Barreau, Christophe Guissard, Audrey Carrière, Yves Martinez, Xavier Descombes, Luc Pénicaud, Jacques Rouquette, Louis Casteilla, Franck Plouraboué, Anne Lorsignol

**Affiliations:** 1 IMFT, Université de Toulouse, CNRS, INPT, UPS, Toulouse, France; 2 STROMALab, Université de Toulouse, CNRS ERL 5311, EFS, INP-ENVT, Inserm U1031, UPS, Toulouse, France; 3 FRAIB, CNRS - FR 3450, Castanet-Tolosan, France; 4 Université Côte d’Azur, INRIA, I3S, Nice, France; 5 ITAV, Université de Toulouse, CNRS USR 3505, Toulouse, France

**Keywords:** Image processing, Image processing, Fat metabolism, Fat metabolism

## Abstract

Adipose tissue, as the main energy storage organ and through its endocrine activity, is interconnected with all physiological functions. It plays a fundamental role in energy homeostasis and in the development of metabolic disorders. Up to now, this tissue has been analysed as a pool of different cell types with very little attention paid to the organization and putative partitioning of cells. Considering the absence of a complete picture of the intimate architecture of this large soft tissue, we developed a method that combines tissue clearing, acquisition of autofluorescence or lectin signals by confocal microscopy, segmentation procedures based on contrast enhancement, and a new semi-automatic image analysis process, allowing accurate and quantitative characterization of the whole 3D fat pad organization. This approach revealed the unexpected anatomic complexity of the murine subcutaneous fat pad. Although the classical picture of adipose tissue corresponds to a superposition of simple and small ellipsoidal lobules of adipose cells separated by mesenchymal spans, our results show that segmented lobules display complex 3D poly-lobular shapes. Despite differences in shape and size, the number of these poly-lobular subunits is similar from one fat pad to another. Finally, investigation of the relationships of these subunits between each other revealed a never-described organization in two clusters with distinct molecular signatures and specific vascular and sympathetic nerve densities correlating with different browning abilities. This innovative procedure reveals that subcutaneous adipose tissue exhibits a subtle functional heterogeneity with partitioned areas, and opens new perspectives towards understanding its functioning and plasticity.

## Introduction

A small but growing amount of data suggests that tissues/organs should be seen as sophisticated ecosystems depending on highly complex but structured interactions between different, organized sets of cells and their micro-environments^1^. Increasing knowledge of precise 3D tissue architecture/structure with cell resolution is now mandatory to progress in the understanding of tissue-organ physiology and pathology. One emblematic tissue is the liver, where the description of the architecture and vascularization of hepatic lobules allowed a better understanding of how different metabolic functions are assumed by their partitioning^2,3^. More recently, studies on pituitary gland revealed an organization in multiple, intermingled networks of major physio-pathological relevance^4^.

Because of the explosive worldwide development of obesity associated with metabolic disorders and the pivotal role played by adipose tissues in such diseases, understanding their biology appears crucial^5,6^. Adipose tissue, as a metabolic-endocrine-immune organ, is well known for its involvement in multiple physiological functions^7–9^. Since Wassermann’s work in 1960, which described the “lobular” structure of adipose tissue using classical 2D photonic microscopy images^10^, very few studies have investigated the architecture of adipose tissues in depth^11–14^. This is largely due to the technological obstacles encountered when attempting to image a whole tissue and manipulate large datasets. Up to now, most of the studies on adipose tissues have focused on the cellular or the molecular scale. A large number of studies have demonstrated that adipose depots differ from each other in terms of “adipocyte biology” including adipokine secretion and/or rate of lipolysis and triglyceride synthesis and that a complex cellular heterogeneity exists within a single fat pad^6,15–21^. However so far there has been no in-depth investigation of the whole 3D anatomy with cellular resolution that established a link between cell partitioning and functional heterogeneity within a single fat depot.

Recently, we made some initial progress in this field with up-to-date imaging that highlighted the existence of two large, distinct areas within the murine subcutaneous fat pad^13^. The first one, located in the core of the pad, contains fat subunits with lobule-like shapes and has inducible brown-like or beige adipocytes, able to express the uncoupling protein-1 (UCP1) after cold exposure^22–24^. The second one, at the periphery of the tissue, does not exhibit any identifiable subunits and is refractory to cold-induced browning. Unfortunately, in this study, automatic 3D segmentation of lobules using commercially available softwares was unsuccessful and systematic analysis of several samples was therefore not possible. This prevented us from studying 3D structures of the subunits and their interconnectivity.

In the work reported here, we developed a new semi-automated method of segmenting structural subunits, based on contrast enhancement, and applied it to 3D imaging of the whole murine subcutaneous fat pad once it had been cleared. Whatever the fluorescent signals used for imaging (high-resolution autofluorescence or vascularization signals), our combination of in-depth imaging and computation revealed that subcutaneous adipose tissue presented a more complex 3D anatomy and greater heterogeneity than previously thought. The 3D subunits that had been identified as ellipsoidal lobules in 2D, displayed a glove-finger and poly-lobular shape. Moreover, our results show that a change of the size of the whole fat pad is accompanied by modification of size of each subunit with no change in their number. Investigation of putative functional relationships between subunits by our segmentation procedure reveals a peculiar organization of these subunits in two clusters. Using laser capture microdissection and immunolabelling experiments, we showed that these two clusters displayed distinct molecular signatures and specific vascular and sympathetic nerve densities associated with different browning abilities.

## Results

### Segmentation of 3D images reveals existence of complex subunits in the core of the fat pad

We decided to image 3 fat pads of quite different sizes in order to take account of a putative variability in our analysis. For each sample, autofluorescence signal acquisition revealed two large regions with macroscopic features as already described in our previous study with another strategy^13^. Subunits could be segmented in the core of the tissue, while no segmentation was possible at the periphery. So, in order to limit the volume of calculations, a first segmentation step was used to separate two large regions: with and without lobular organization. Volume quantification of both regions of the fat pad revealed that the proportion corresponding to the core (previously named SLA, for Segmentable Lobule Area^13^) was similar in all samples (20.1 ± 1.9% of the total volume of the fat pad) with a mean volume of 19.5 ± 8 mm^3^ (Table 1) (n = 3).

**Figure 1:**
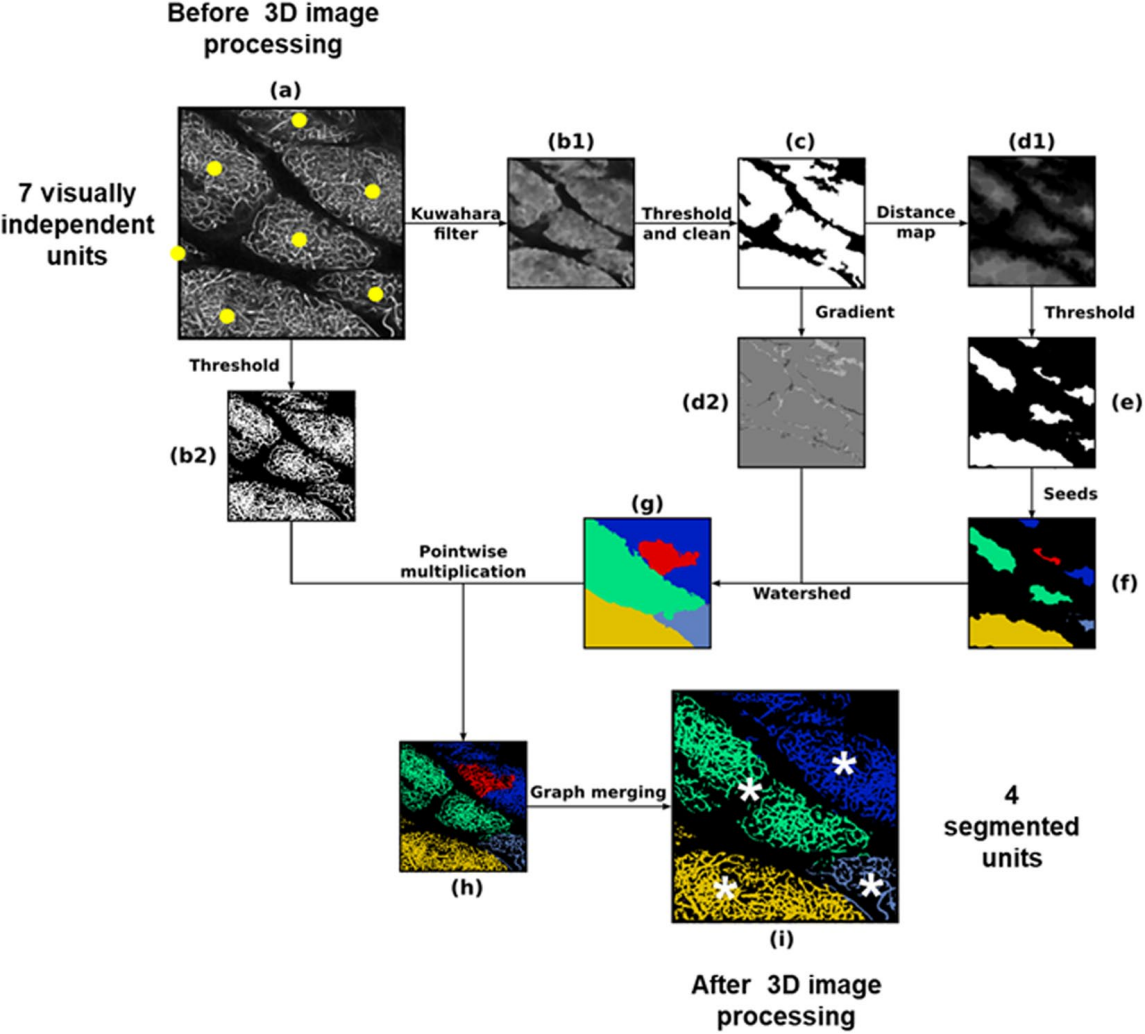
Schematic illustration of the 3D image post-treatment work-flow. Each sub-figure represents the result of one step of the algorithm. The links between them describe the operations needed to pursue one step after another. (**a**) Downscaled original image before processing, (**b1**) image filtered using a customized Kuwahara filter (each pixel is replaced with the mean of the means/variances ratio evaluated at neighbouring boxes), (**b2**) downscaled original image binarized using a simple threshold, (**c**) filtered image binarized using a simple threshold and cleaned using mathematical morphology closure treatments to fill gaps and remove smallest “islands”, (**d1**) result of distance map, (**d2**) gradient image using Sobel method, (**e**) binarized image using simple threshold, (**f**) seeds for watershed obtained by computing connected components, (**g**) watershed image obtained using flooding method, (**h**) pre-segmented image obtained from original downscaled binarized image and watershed image by point-wise multiplication (result from (**b2**) and (**g**)), (**i**) final segmented image after a graph merging method based on evaluation of contact surface between relative to mean total surface for every pair of pre-segmented subunits. Each colour codes one 3D segmented unit. One can note that the segmentation procedure finally detects four different units (white asterisks, picture “i”) whereas human eye would distinguish seven units (yellow points, picture “a”).

**Figure 2:**
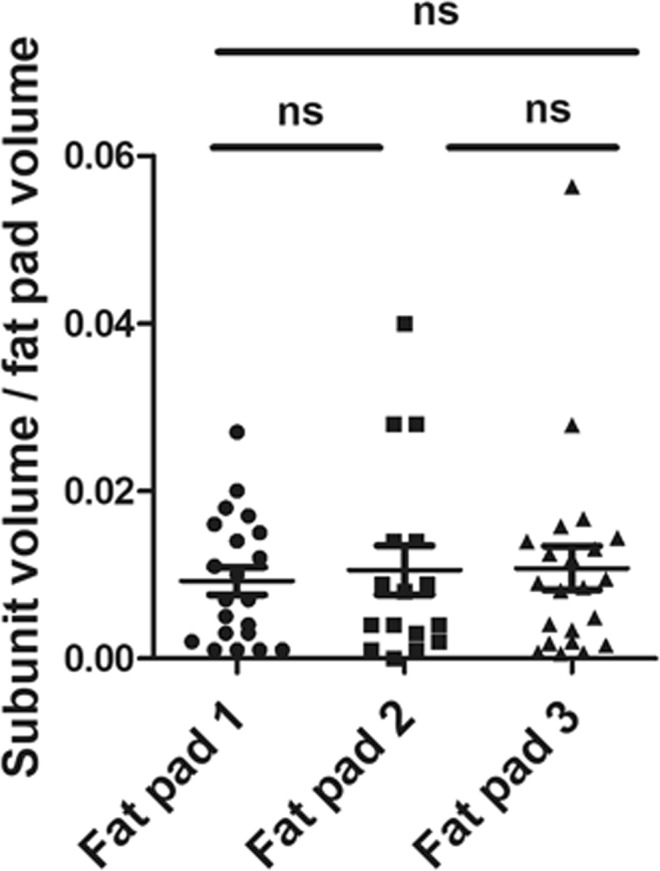
Whatever the size of the fat pad, number and relative volume of segmented adipose subunits are similar. For the three samples, the volume of each segmented subunit was normalized to the volume of the whole fat pad.

We then developed a segmentation procedure based on contrast enhancement, whose robustness was investigated and discussed in Supplementary Information (Fig. S1). This segmentation procedure described in Fig. 1 and applied to SLA, allowed 15 to 21 fat subunits to be identified (Table 1). Their size varied from less than 0.04 to more than 8 mm^3^ depending on the volume of the sample (Table 1) but, strikingly, after normalization to the volume of the whole fat pad, no significant difference was observed in the subunit relative volume (Fig. 2). We verified that our segmentation procedure was not restricted to autofluorescence signal by labelling vessels with *in vivo* lectin injection and applying similar automatic segmentation procedure. Supplementary Fig. S2 shows that analogies can be found between segmented subunits obtained on a given tissue sample, with the two distinct signals.

**Table 1:** Morphometric parameters of the three mouse inguinal fat pads.

	inguinal fat pad no. 1	inguinal fat pad no. 2	inguinal fat pad no. 3	Mean ± SEM
Fat pad volume (mm^3^)	38.58	95.89	145.54	93.3 ± 30.9
Volume of the Non-Segmentable Lobule Area (mm^3^)	31	79.55	110.97	73.5 ± 23.3
Volume of the Segmentable Lobule Area (SLA) (mm^3^)	7.58	16.34	34.57	19.5 ± 8
ratio SLA/fat pad volume (%)	19.6	17	23.8	20.1 ± 1.9
number of segmented subunits	20	15	21	
min volume of subunits (mm^3^)	0.038	0.042	0.093	
max volume of subunits (mm^3^)	1.052	3.825	8.196	
mean volume of subunits (mm^3^)	0.361 ± 0.28	1.021 ± 1.11	1.57 ± 1.78

**Figure 3:**
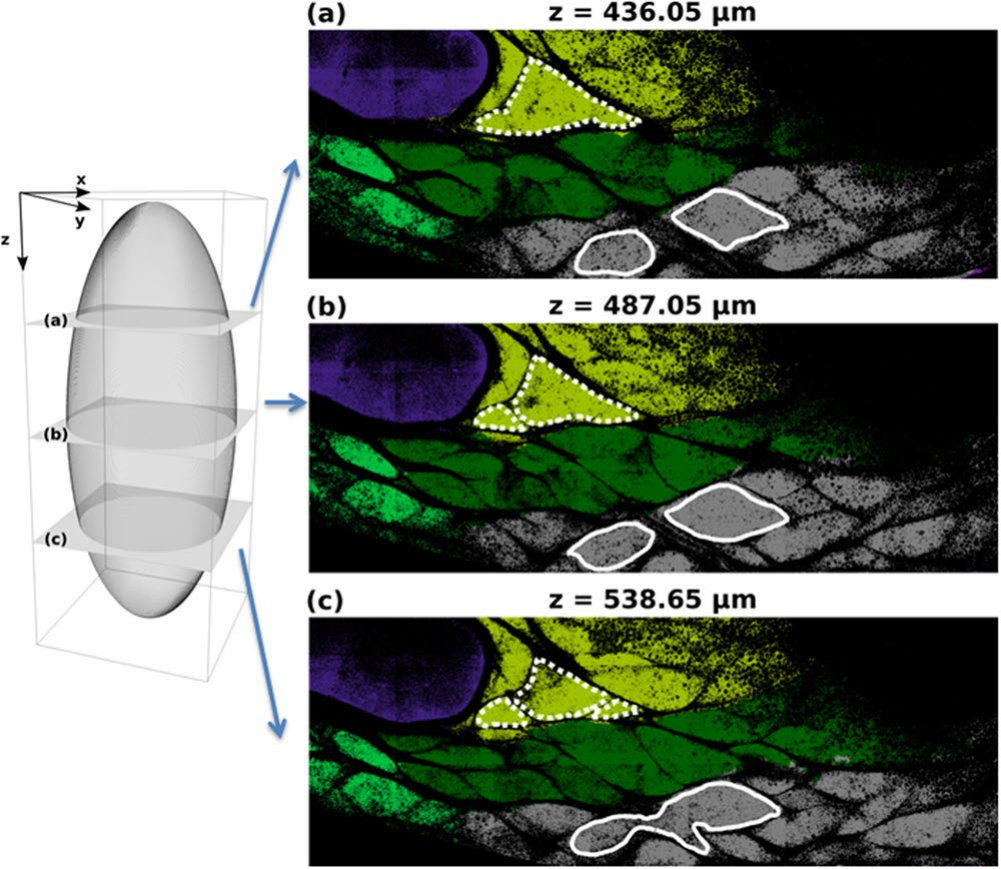
Illustration of misleading 2D slicing on subunit identification. Left panel provides the schematic representation of three different slices (**a**–**c**) chosen along the z direction. Right panel illustrates tissue optical section chosen at three z positions: (**a**) z = 436.5 µm, (**b**) z = 487.05 µm, (**c**) z = 536.65 µm. The colours code the 3D segmented subunits given by the automatic post-treatment procedure explained in the Materials and Methods section and illustrated in Fig. 1. The external contour of two different subunits is delineated with white (respectively, dotted and continuous) lines. Depending on the slice (**a**–**c**), the 2D optical section of each subunit could appear disconnected whereas they are, in fact, fully connected in 3D.

Qualitatively, our segmentation revealed complex shapes for the identified subunits. This is in contrast with the classical 2D view of adipose tissue, which shows a superposition of simple ellipsoidal lobules. From the analysis of various slices in the z direction, Fig. 3 illustrates the 3D connection between apparently individual, small lobular subunits leading to wider, more complex, poly-lobular subunits. The green mono-lobular subunit surrounded by the dotted line in Fig. 3a splits into two and then three distinct subunits when different slices are observed (Fig. 3b,c). Similarly, the two grey subunits with continuous contours in Fig. 3a are closer together in Fig. 3b, and merge into a single subunit in Fig. 3c. The shapes of these inter-digitated subunits were distinct from one other (Supplementary Movies 1 and 2) but always with a preferential direction in the X-Y plane (Supplementary Movie 3). In the rest of the manuscript, we will use the term “subunit” to refer to these poly-lobular structures.

### Investigation of location and inter-connection of subunits suggests the existence of two macro-domains inside the Segmentable Lobules Area

**Figure 4:**
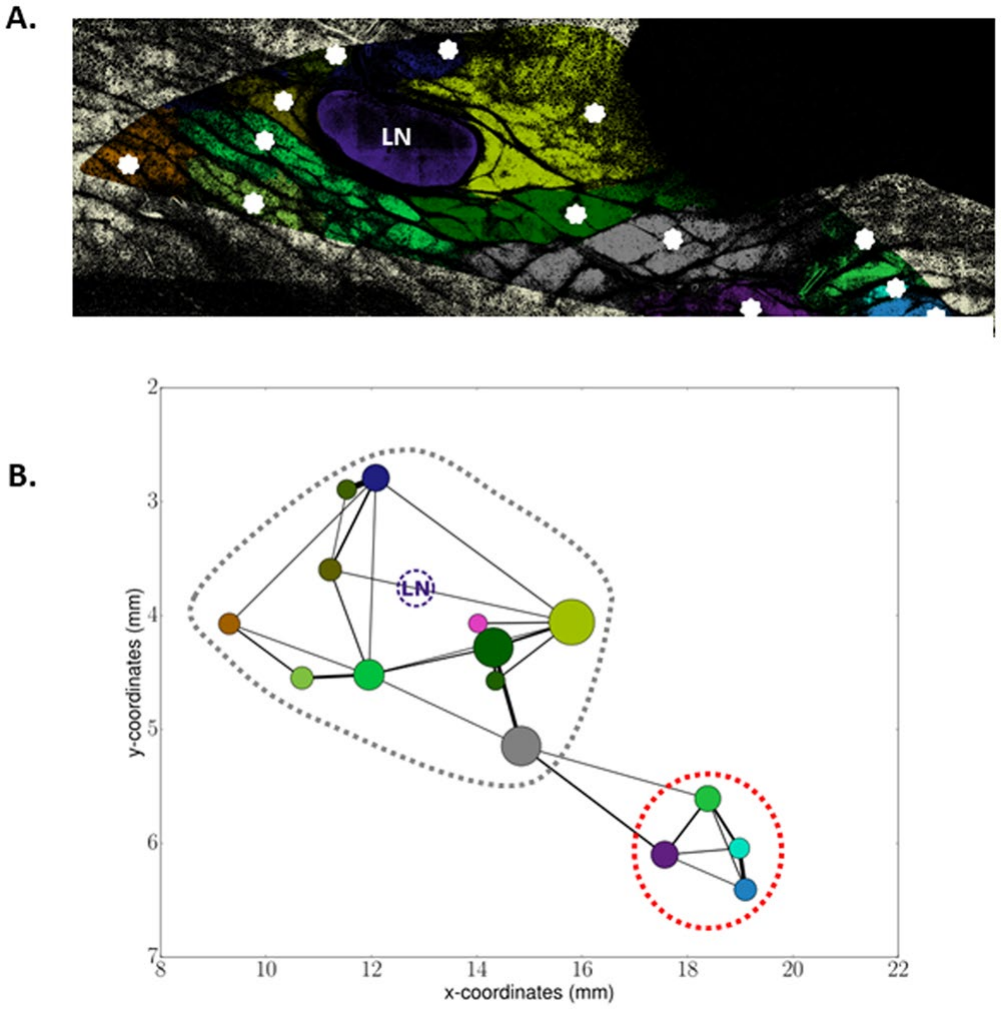
Segmentation reveals two clusters of interconnected polylobular subunits. (**A**) Representation of a segmented autofluorescence signal image for one slice. The image has been cropped to focus on the zone where subunits are identifiable. Non Segmentable Lobule Area is displayed in light yellow (on the borders) whereas Segmentable Lobule Area (SLA) is composed of the coloured subunits. The large violet ovoid structure is the lymph node (LN). Each white star corresponds to one segmented subunit. (**B**) 2D Graph representation of the subunit locations, connections and sizes in the segmented zone. The (x, y) coordinates indicate the position within the fat pad shown in (**A**); x_0_,y_0_ corresponding to the apical extremity of the fat pad. The positions of the nodes of the graph, i.e. the centre of each coloured circle, are those of the subunit barycenters. The radius of the circle is proportional to the volume of the subunits. Colour of each subunit is the same as in the colour map chosen in (**A**). The links between two nodes are computed as the ratio of the contact surface between two subunits to their mean total surface. Thus, the larger the link is, the stronger is the connection between the two subunits. Note that the pink and the small bottle-green subunits in the graph correspond to small subunits that do not appear on the slice shown in (**A**). The grey and red dotted circles illustrate the two clusters of well-connected subunits.

A direct consequence of highlighting these complex shapes was that their volumes were seen to be bigger than those suggested by classical 2D images as illustrated in Fig. 4A, where only 13 subunits can be observed (represented by distinct colours and white stars), distributed inside the core region of the fat pad. 3D mapping of these subunits does not reveal any predetermined pattern. However, at a larger scale, a preferential alignment of segmented subunits from the left to the right sides (corresponding to the apical-to-groin axis of the fat pad) could be observed in the vicinity of the lymph node (Fig. 4A).

The 3D structure and inter-connection of all the subunits was analysed in greater detail using an unbiased automated computation of the 3D connections of each segmented subunit from image analysis. Functional links between subunits were quantified by considering that the number of common voxels at the frontier of two distinct segmented subunits indicated the presence of neighbouring cells that were able to exchange biological signals and molecules from one entity to the other. The graph of the resulting links for fat pad number 2 is shown in Fig. 4B. This graph not only illustrates the heterogeneity of the subunit volumes, since each circle is proportional to the volume of the corresponding subunit, but also describes how these subunits are connected to each other in 3D. Figure 4B clearly shows that, near the lymph node (LN), the subunits are closely connected, forming a “cluster” of well-connected subunits (highlighted by the grey dotted circle). Furthermore, four subunits well connected into a second, smaller cluster are observed near the groin (red dotted circle). This specific architecture of connections is again found in fat pad number 3 (Supplementary Fig. S3A). In the third inguinal fat pad, six subunits located on the right are, again, more strongly connected to each other (illustrated by larger links) than with the other subunits (supplementary Fig. S3B). Taken as a whole, the analysis of these 3D connections revealed another scale of the tissue anatomy, with a spatial organization of the subunits in two macro-domains, one including 13 ± 1 subunits located near the lymph node and the other composed of 5 ± 1 subunits located at the extremity of the fat pad. The boundary between these two macro-domains was located 3.7 to 4 mm from the lymph node, obviously depending on the specific size of the core region which varied (Table 1).

### The two clusters revealed by the graph representation display distinct molecular, vascularization and innervation profiles

**Figure 5:**
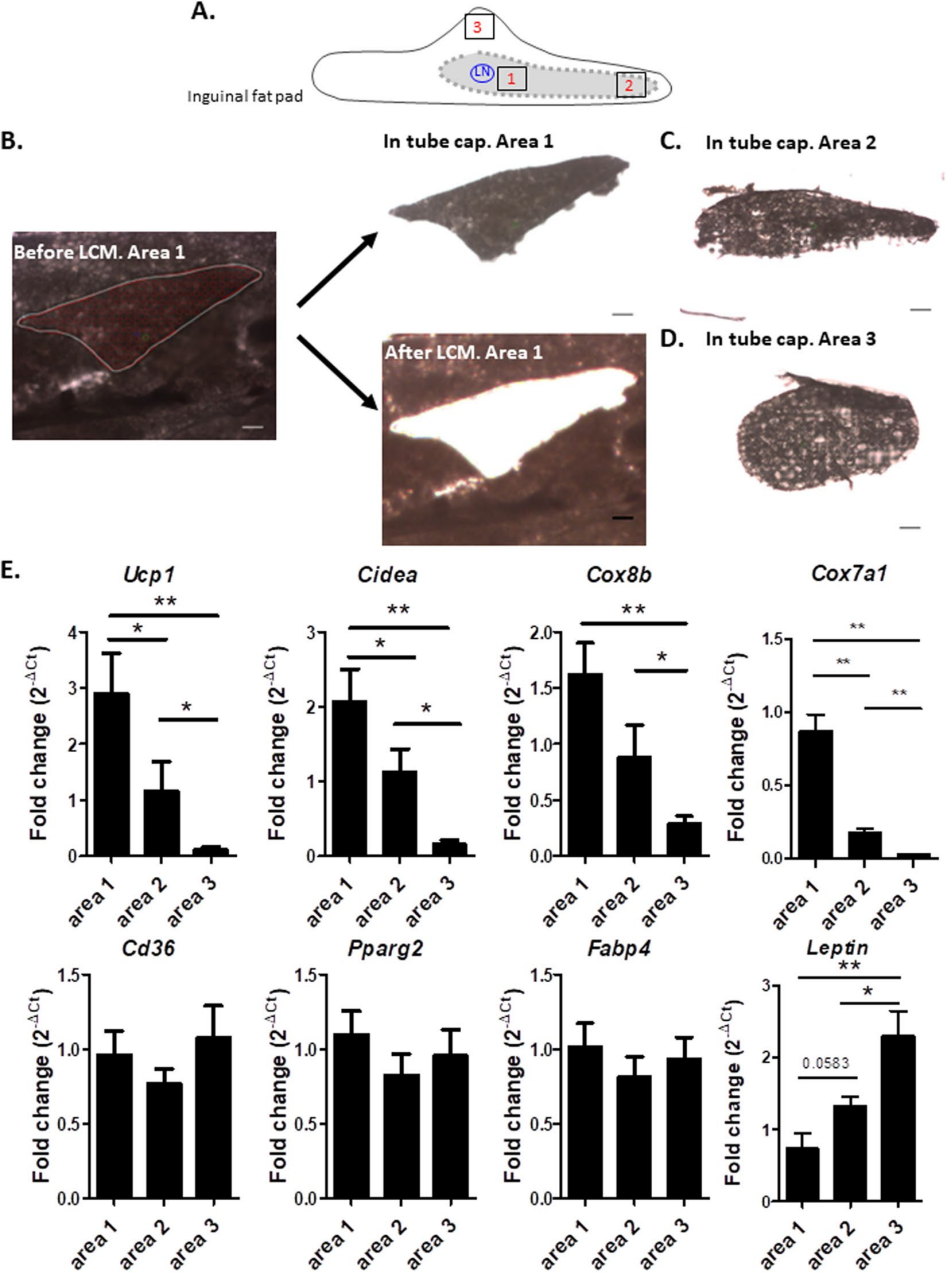
The two clusters display distinct molecular signatures. (**A**) Sketch illustrating where laser capture microdissection was performed. (**B**) Example of Laser Capture Microdissection (LCM) of area 1 of inguinal fat pad. Scale bar corresponds to 100 µm. Left panel: Selection of the area to be cut by LCM. Right panel top: Dissected area captured on the cap. Right panel bottom: Tissue section after the cut elements have been dissected. (**C**) Example of LCM of area 2. (**D**) Example of LCM of area 3. (**E**) Gene expression analysis in areas 1, 2 and 3 (shown in the sketch) of inguinal fat pad of C57Bl6 mice exposed to temperature of 22 °C. After LCM, total RNA was isolated from each area and assayed for mRNA levels of *Ucp1, Cidea, Cox8b, Cox7a1, Leptin, Cd36, Pparg2* and *Fabp4* by RT-QPCR. (n = 7, 3 and 5). *P < 0.05 and **P < 0.01 comparison between areas.

As recently demonstrated by us and others^13,14^, the core region of the inguinal fat pad has great browning ability compared to the periphery. To investigate whether the two previously identified clusters could delineate domains with distinct molecular signatures related to metabolism and browning, we laser-microdissected several small pieces of adipose tissue near the lymph node (area 1, corresponding to the largest cluster) or near the groin extremity (area 2, corresponding to the second cluster). As controls, laser micro-dissections were also performed outside these clusters, i.e. at the periphery of the fat pad (area 3) (Fig. 5A). Figure 5B–D shows that marked areas were efficiently laser micro-dissected from the inguinal fat pad and captured. Real-time quantitative PCR analysis performed on RNA extracted from the different micro-dissected areas of inguinal depots harvested from C57Bl6 mice maintained at 22 °C showed that *Ucp1* and *Cidea* mRNA levels, encoding two proteins specifically expressed by brown-like adipocytes, were significantly enriched in area 1, close to the lymph node, compared to area 2 (Fig. 5E). Similar trend was observed for other brown-like adipocytes markers^25,26^ including *Cox8b* and *Cox7a1* (Fig. 5E). The opposite result was obtained for *leptin*, a marker of white adipocytes. As expected, area 3 (Fig. 5A) containing adipocytes refractory to the browning process^13^, showed the lowest levels of thermogenic gene expression and the highest levels of *leptin*. Expression of adipogenic genes non-specific to white or brown-like adipogenesis, such as *Cd36*, *Pparg2* and *Fabp4* was not significantly different among the three micro-dissected areas.

Brown-like adipocytes are characterized by large number of mitochondria, which support their thermogenic properties. Their biological activity is closely linked to sympathetic innervation and vascularization to control their thermogenic functions and to ensure a sufficient supply of nutrients and oxygen and to handle heat export. We thus estimated mitochondrial equipment with TOM20 immunostaining and observed a heterogeneous distribution of mitochondria through the SLA region, with a clear enrichment of mitochondria in area 1 (Supplementary Fig. S4A). Higher density of tyrosine hydroxylase immunoreactive fibers in area 1 compared to areas 2 and 3 was also observed, suggesting wider sympathetic innervation within the region close to the lymph node (Supplementary Fig. S4B,C). Finally, computation of the vascular density on specific regions of interest revealed higher vascular density in area 1 than in area 2 and the periphery showed the lowest values (Supplementary Fig. S4D).

### The two clusters display differential response to cold-induced browning

**Figure 6:**
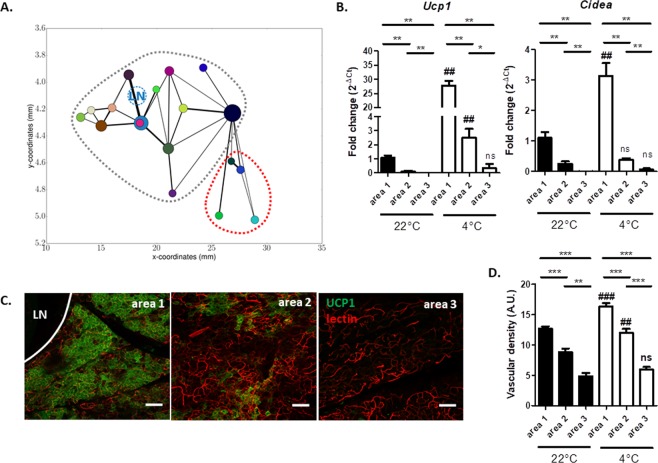
The two clusters present distinct browning abilities upon cold exposure. (**A**) 2D Graph representation of the subunit locations, connections and sizes in the segmented zone of inguinal fat pad from mice exposed to 4 °C for 48 hours. (**B**) *Ucp1* and *Cidea* expression analysis in areas 1, 2 and 3 of inguinal fat pad of C57Bl6 mice exposed to 22 °C and 4 °C (n = 6 animals for each area and each conditions) for 48 hours. (**C**) Immunostaining of UCP1 (green) and acquisition of lectin signal (red) performed on areas 1, 2 and 3 of inguinal fat pad sections of C57Bl6 mice exposed to 4 °C for 48 hours. LN, lymph node. Scale bar corresponds to 100 µm. (**D**) Investigation of vascular density by quantifying lectin labelling on binarized images acquired on inguinal fat pad section of C57Bl6 mice exposed to 22 °C or 4 °C. (n = 10 ROI for each area and each conditions). **p < 0.01, ***p < 0.001 comparison between different areas at the same temperature. ^##^p < 0.01, ^###^p < 0.001 comparison between 22 °C and 4 °C for a given area.

To determine whether the distinct molecular profiles, the mitochondrial apparatus and the nervous and vascular networks were predictive of browning abilities under noradrenergic stimulus, C57Bl6 mice were exposed to 4 °C for 48 hours. Our automatic segmentation procedure applied on two samples revealed that cold exposure did not seem to affect dramatically the relative volume, the number, and the interconnectivity of subunits (Figs 6A and Supplementary Fig. S5). Figure 6B shows that area 1 contained higher *Ucp1* and *Cidea* mRNA levels than areas 2 and 3 after cold exposure. Consistently with molecular analysis, higher UCP1 immunostaining was observed in area 1 than in area 2 after cold exposure, area 3 being devoid of brown-like adipocytes (Fig. 6C). Finally, quantification of lectin labelling revealed an increase of vascular density in areas 1 and 2 after cold exposure without changing the differential pattern between areas 1, 2 and 3 observed at 22 °C (Fig. 6D).

## Discussion

In a previous study, we demonstrated that this depot was spatially organized in two large regions with sub-domains associated with a compartmentalization of browning ability^13^. However, the computational approach used in that study did not allow complete identification of lobules, which were classically seen as ellipsoidal clusters of adipocytes. Therefore, here, we developed a dedicated and novel 3D tissue-scale image analysis workflow in order to provide objective segmentation of tissue organization. Altogether, our results show that the whole subcutaneous fat pad possesses a complex spatial and functional organization.

Our developed segmentation procedure, albeit based upon classical image analysis tools, has specific and highly technical features, leading to a customized and efficient C++ code. It allowed semi-automatic treatment of a mosaic of 3D images containing several hundred blocks, while keeping memory use low thanks to an adequate pyramid strategy. Furthermore, the parameterization of several steps of the workflow (e.g. the filtering steps) has undergone time-consuming calibration tests and procedures so as to be able to deliver robust and satisfactory segmentation results. The resulting segmentation strategy allows semi-automated treatment to be achieved for large-scale 3D tissue images, which would not have been possible with manual handling. Image segmentation is a long standing topic in image analysis, of particular relevance to biomedical imaging communities. The workflow proposed in this article is original as it has been dedicated to the specific task at end but follows conventional pipelines. Deep-learning methods disrupt the field of image segmentation because they permit robust, performant, and fully automated workflows when possible^27–30^. These methods outperform traditional approaches when it is possible to train the algorithms on huge databases onto which ground truth segmentation is available but are also often a distressing step on large 3D images. Regarding the topic of interest here, the building of a training data-base of 3D fat-pad lobules would have been not only a costly and intensive issue, but would have also required a huge amount of 3D images not possible to provide in our case.

Tissue 3D confocal imaging using either autofluorescence or blood vessels labelling combined to semi-automated segmentation procedure developed here, allow us to assess functional fat pad tissue heterogeneity in physiological and pathological contexts. Our results confirmed that fat subunits are only present in the core region, and also revealed that these subunits display complex and unexpected 3D inter-digitated poly-lobular shapes. In contrast to other lobular structures found in the liver^31^ or pancreas^32^, where single but repetitive structural patterns have been described, even if performed on only three mice, our 3D analysis of the whole pad revealed different shapes and volumes for the segmented subunits of fat, suggesting that structural heterogeneity may support functional heterogeneity.

Despite differences in shape and size, the number and inter-connectivity of subunits were similar from one fat pad to another. Putative differences according to age, sex or mice strains still need to be unravelled. Between fifteen and twenty subunits were segmented and graph representing their connection revealed two macro-domains of well-connected subunits: a large one near the lymph node, and a smaller one near the groin. Microdissection experiments and immuno-labelling revealed different expression levels of browning genes and mitochondrial apparatus in these two sub-domains than in the periphery. As expected, a high expression of *leptin* with almost no expression of *Ucp1* or *Cidea* was observed at the periphery of the inguinal fat pad, while the opposite was seen in the core of the tissue^13^. Microdissection inside the two sub-domains revealed that the cluster closer to the lymph node displayed higher *Ucp1* and *Cidea* mRNA levels and a greater number of mitochondria than the more distant one. Consistently with these results, the highest browning process in response to cold challenge was observed around the lymph node, further highlighting the existence of heterogeneity between the subunits. Of note, high UCP1 and lectin labelling were also observed in the other side of the lymph node (data not shown), suggesting no obvious difference in browning ability of subunits within the same cluster. This opens up the question of the physiological relevance of close association between the lymph node and the subunits with highest browning ability. Even if such neighbourhood is consistent with data reporting links between immune cells and browning process^33^, we cannot exclude that local thermogenesis resulting from heat generating brown-like adipocytes might support the functionality of the adjacent lymph node and immune responses. Finally, a strong parallel between vascular and sympathetic nerves densities and browning ability could be observed not only between the periphery and the core region but also inside the core, between the two identified sub-domains. This reinforces the idea that vasculature and sympathetic innervation could be predictive of browning ability^14,34^.

A graph representation of the spatial organization was used to decipher the relationship between functional subunits. Although such representations are widely used in many areas, not only for describing web and social networks but also for complex urban and geographical spatial structures, they are rarely used in the context of soft-tissue structural analysis^35–38^. Nevertheless, they overcome the limitations of 2D imaging to identify and illustrate the true connection between two 3D subunits, and highlight some specific, non-regular, organization levels, such as preferential couplings. This type of graphic representation, when applied to segmented adipose subunits revealing two clusters of well-connected subunits, opens up the question of the physiological relevance of this architecture. Since similar segmentation results were obtained with autofluorescence and blood vessels labelling, we can speculate that the subunits macro-domains overlap the vascular network and thus, as in the brain^35^, couplings between subunits correspond to preferential blood perfusion. Moreover, as the browning areas are located along the main vasculature^13^, the two clusters could be thus perfused faster than the periphery, thus questioning the partitioning of the substrate flux among the fat pad as demonstrated in the liver^2^. Unfortunately, this question cannot be addressed with the present segmentation and further efforts are needed to model vasculature and its flow. Moreover, network organization seems to be common to endocrine tissues such as the pancreas, pituitary gland or more diffuse endocrine organs such as the gut^39–41^. In these cases, the specific cell organization plays a key role in the control of hormone secretion^4,42^. We can thus also speculate that this peculiar organization of the inguinal fat pad can control at least some adipocyte secretions that, in turn, can define specific micro-environments for other cells such as immune or stromal cells.

Taken together and even if the results have to be confirmed on a larger number of samples, our segmentation procedure reveals invariant meso-anatomy features of the inguinal fat pad, including the number of subunits and the geography of the network linking all of them, and thus constitutes a first step to study metabolic and/or cellular compartmentalization within the adipose tissue. This observation is encouraging for the extension of the proposed approach to a more systematic investigation of how these patterns appear during development or re-organize in different physiological or pathological conditions. We can speculate that the peculiar and complex 3D anatomy of fat pads described in this study controls the numerous endocrine secretions of this tissue^7,8^ as well as nutrient delivery and heat dissipation from the tissue thus defining specific “niches”. The fact that segmented fat subunits are not biologically equivalent emphasizes the need to better characterize the fine physiological and physio-pathological anatomical and structural organization of adipose tissue. Investigations restricted to core versus peripheral regions of the inguinal fat pad is now not sufficient to understand the precise physiology of this depot (and its probable impairment in a pathological context) and thus cooperation among cells within the tissue. Thus it is now mandatory to study fat subunits and the functional links between them. Since a microvascular network is likely to support these functional connections between fat subunits, investigation of blood vessel architecture and perfusion should be helpful in this field.

## Materials and Methods

### Animals

Experiments were performed on 6- to 8-week-old male C57BL/6J mice (Harlan Laboratories), housed in a controlled environment (12 h light/dark cycle at 22 °C or 4 °C). Mice were group-housed (3 or 4 per cage) with free access to food and water in accordance to guidelines of the European Community Council (2010/63/UE). Cold exposure lasted 48 hours. All experimental procedures were done in compliance with European regulations for animal experimentation. The authors have received requested approval from their Institutional Ethic Committee (CEA 122) and from the Ministry of National Education, Higher Education and Research (Protocol Reference: 8326-2016122308408946 v2) for all the experiments performed.

### Whole Tissue imaging

Fed mice were anaesthetized by intraperitoneal injection of ketamine/xylazine mix and submitted to intra-cardiac perfusion with 4% paraformaldehyde solution. To visualize the vasculature, Rhodamine labelled Griffonia (Bandeiraea) Simplicifolia Lectin I (Eurobio Abcys) was injected into the orbital sinus before sacrifice. Inguinal fat pads (right or left side) were removed, oriented (apex versus groin), post-fixed at 4 °C overnight and kept in PBS at 4 °C. Tissues were then embedded in 1% agarose before being dehydrated by graded series of ethanol incubations and then cleared by incubation in benzyl alcohol-benzyl benzoate solution (BABB, 1:2 vol:vol ratio Sigma Aldrich) as already described^13^. Imaging of cleared tissues was performed using a Confocal Laser Scanning microscope (LSM510 NLO-Carl Zeiss, Jena, Germany) as previously reported^13^. Because of the size of the sample, multiple positions were acquired with an overlap of 10% and a z-stack was performed. Final images were obtained by stitching all the acquired positions using image metadata Grid/collection stitching plugins of Fiji software^43^. Autofluorescence and rhodamine signals were collected with 500–530 nm and 560–615 nm band-pass filters respectively. Standardized conditions for pinhole size, and for gain and offset (brightness and contrast), were used for image capture.

### 3D Image processing

The hereby described image processing steps are fully performed in 3D although the image size along the z dimension is smaller than in the horizontal plane. A preliminary step was used to separate regions with and without lobular organization as already suggested^13^. This preliminary step consists in filtering, and thresholding the image so as to be able to segment, i.e. delineate, the Segmentable Lobule Area (SLA) as illustrated in Fig. 4a. Then, the rest of the image analysis procedure is dedicated to the segmentation of the lobules in the 3D image of the SLA, i.e. finding a structural 3D partition to be able to distinguish the frontiers of distinct subunits. A custom C++ code was developed to segment lobules in 3D images from the autofluorescence signal. The lobule segmentation procedure is described in Fig. 1 and more extensive details are also provided in the appendix. Here, we present a summary of the segmentation procedure. Firstly, the dense entities were homogenized and their frontiers preserved using a non-linear mask, as illustrated in Fig. 1b1. Then, the image was binarized and small holes were filled (Fig. 1c). In order to obtain separated entities, the distance map of the binarized image was computed (Fig. 1d1 followed by a binarization (Fig. 1e) and a computation of connected components (Fig. 1f). In parallel, the gradient of the binarized image was computed (Fig. 1d2) to set the borders of the segmented subunits. We then multiplied (pointwise multiplication) the original grayscale image (Fig. 1b2) by a watershed algorithm (Fig. 1b2) (using seeds from connected components and borders from the gradient), leading to a pre-segmented image illustrated in Fig. 1h. Finally, to correct artefacts from holes in the gradient borders, the pre-segmented entities were merged, based on their contact surface to total surface ratio. The final result of this segmentation procedure is illustrated in Fig. 1i. It should be pointed out that this procedure is semi-automatic and parameters must be chosen accordingly. All parameters are reported in the Additional Information section.

### Post-processing quantifications

Once the 3D images had been processed, the volume of each segmented lobule was defined by computing the total number of voxels of the watershed mask, which was translated into a physical volume. The contact surface between each pair of segmented lobules was also computed as the voxels having a distinct label from their neighbours and whose labels corresponded to those of the pair of lobules considered.

Since lectin labelling was localized in vessel walls, we further quantified the average grayscale density in this modality as it provided quantitative information on vessel density in various regions and could be considered as a proxy variable for the vascular density. This was evaluated in both the non-segmented (periphery of the fat pad) and the segmented (SLA) region after subtraction of the lymph node. We also computed the proxy of the vascular density on specific 3D regions of interest on grayscale images.

### Immunohistochemistry

The inguinal fat pad of fed mice was fixed in paraformaldehyde 4% overnight before being cut into 300 µm sections using a vibratome (Campden). Sections were incubated in blocking solution (2% normal horse serum and 0.2% triton X-100 in PBS) at room temperature before being incubated for 24 h at room temperature with primary UCP1 (1:5000, kindly provided by D. Ricquier), TOM20 (1:400, Santa Cruz, sc-11415) or TH (1:750, Abcam AB1542) antibodies. After overnight incubation at 4 °C with Alexa 488- or Alexa 555-conjugated secondary antibodies (Life Technology), imaging was performed using a confocal laser scanning microscope (LSM880 NLO, Carl Zeiss). Quantification of sympathetic innervation and vascularization was performed on binarized images obtained after TH or lectin labelling. At least ten regions of interest (ROI) of the same size were randomly selected in areas 1, 2 and 3 from one inguinal fat pad based on tissue autofluorescence. Sympathetic nerve or vascular density was expressed as the ratio of, respectively, TH- or lectin-labelled pixels to the total number of pixels per ROI by using Fiji software.

### Laser capture microdissection (LCM) and gene expression analysis

Inguinal fat pad harvested from fed mice was fixed in methanol (−20 °C, overnight) and placed in a plastic Cryomold filled with tissue freezing compound (Tissue-Tek® OCT), frozen in isopentane and stored at −80 °C before being sliced into 50 µm sections using a cryostat (MICROM HM 560V). Sections were placed on membrane coated slides, immersed in chilled 70% ethanol for 30 sec and then rinsed in water for 15 sec. Slides were then immediately immersed in graded series of ethanol (70, 95 and 100%, for 30 sec). Tissue sections were cleared in xylene for 2 min and air dried for 5 min. All steps were performed in RNAse free conditions. LCM was carried out under 10X magnification microscopic visualization using the ARCTURUS XT apparatus (ArcturusXT™ microscope system). Dissected cells were collected in the collecting tube cap filled with 50 µl of lysis buffer (Arcturus^TM^ PicoPure^TM^ RNA isolation Kit). Total RNA was extracted according to the manufacturer’s instructions. After the control of RNA quality using an Agilent 2100 Bioanalyzer, 30 ng of total RNA was reverse-transcribed using the Superscript Vilo cDNA synthesis kit (Life technology). Quantitative real-time PCR was performed using SYBR Green PCR Master Mix (Applied Biosystem) and 0.3 mM primers on a Viia7TM (Applied Biosystem) instrument. Relative gene expression was determined using the 2-ΔCT method and normalized to 18 s. The primers are listed in Table 2.

**Table 2:** List and sequences of primers.

Target name	Forward Primer	Reverse Primer
18 *s*	AGTCCCTGCCCTTTGTACACA	CGATCCGAGGGCCTCACTA
*Ucp1*	GACCGACGGCCTTTTTCAA	AAAGCACACAAACATGATGACGTT
*Cidea*	CTAGCACCAAAGGCTGGTTC	CACGCAGTTCCCACACACTC
*Leptin*	ACCATTGTCACCAGGATCAA	ACCCTCTGCTTGGCGGATA
*Pparg2*	AGTGTGAATTACAGCAAATCTCTGTTTT	GCACCATGCTCTGGGTCAA
*Fabp4*	GATGCCTTTGTGGGAACCTG	GCCATGCCTGCCACTTTC
*Cd36*	GATGTGGAACCCATAACTGGATTCAC	GGTCCCAGTCTCATTTAGCCACAGTA
*Cox8b*	GAACCATGAAGCCAACGACT	GCGAAGTTCACAGTGGTTCC
*Cox7a1*	CAGCGTCATGGTCAGTCTGT	AGAAAACCGTGTGGCAGAGA

### Statistical analysis

All results are expressed as means ± S.E.M. A non-parametric test (Mann-Whitney) was used to calculate final *P*-values using GraphPad Prism software. Differences among groups were considered significant at *P* < 0.05.

## Supplementary information

Supplementary Information

Movie 1

Movie 2

Movie 3
