# Supplementary material for: 3D analysis of the whole subcutaneous adipose tissue reveals a complex spatial network of interconnected lobules with heterogeneous browning ability
Source: Sci Rep. 2019 Apr 30;9:6684. doi: 10.1038/s41598-019-43130-9 (PMC6491608; doi:10.1038/s41598-019-43130-9)

SUPPLEMENTARY INFORMATION:

TITLE: 3D analysis of the whole subcutaneous adipose tissue reveals a complex spatial network of interconnected lobules with heterogeneous browning ability

AUTHORS:

Dichamp Jules[1]

Barreau Corinne[2]

Guissard Christophe[2]

Carrière Audrey[2]

Martinez Yves[3]

Descombes Xavier[4]

Pénicaud Luc[2]

Rouquette Jacques[5]

Casteilla Louis[2]

Plouraboué Franck[1]

Lorsignol Anne[2]

AFFILIATIONS :

1. IMFT, Université de Toulouse, CNRS, INPT, UPS, Toulouse, France

2. STROMALab, Université de Toulouse, CNRS ERL 5311, EFS, INP-ENVT, INSERM U1031, UPS, Toulouse, France

3. FRAIB, CNRS – FR 3450, Castanet-Tolosan, France

4. Université Côte d'Azur, INRIA, I3S, Nice, France

5. ITAV, Université de Toulouse, CNRS USR 3505, Toulouse, France

Details of the segmentation procedure

The stitched image mosaic of an entire tissue acquisition can be as large as 12000x12000x300 pixels, leading to images of several tens of gigabytes. Each elementary acquisition volume ranges between 256x256x300 and 1024x1024x300 voxels and many hundreds are assembled within a mosaic. For this reason, the automatic treatment of the image is much more easily handled with a customized and scriptable home-made C++ code, which we developed using the Cimg library (http://cimg.sourceforge.net/index.shtml).

We first segmented the two tissue regions where there was no “lobular organization” as opposed to the one where lobules could be segmented [1]. A mean 3D filter from Fiji software was set up, whose size could vary depending on the sample. The mean 3D filter was chosen to be significantly larger in the x and y directions than in the z direction to take account of the anisotropy of the tissue in the z direction. Then, a simple thresholding of the graylevel was performed. Since the signal was much lower in peripheral regions than in the core region it was accentuated by using the 3D filter. After this thresholding-segmentation step, the smallest connected components were removed. Finally a convex-hull algorithm using the 3D mesh voxelizer program binvox (http://www.patrickmin.com/binvox/) was used to delineate the core region inside which the lobule segmentation was to be performed.

In order to objectively identify the organization of the 3D lobules, we designed a specific work-flow consisting of the eight steps outlined in Figure 1

a) The stitching was performed with free software [2,3] with a 10% overlap between the adjacent volume of the mosaic and based upon maximum correlation. A pyramid of images with three hierarchical levels was also created so as to save memory allocation in some parts of the work-flow.

b) This step was decomposed into b1 and b2. In b1) the filtering step was adapted. Because of the large heterogeneity of the image texture within each lobule (see Figure 1a) which, in many cases, is as large as the amplitude of the background noise, there was a

need to find an adapted filter which, on one the hand, would smooth graylevels inside the lobules, while, on the other hand, preserving or even improving the contrast at their edges, so as to help their further segmentation. For these reasons, step b1) is crucial, with the additional requirement that image inhomogeneity associated with the background illumination is dealt with. Various methods such as variational or level-set methods and texture filtering could have been used in this first step [4]. We used an adapted Kuwahara texture filter, which allowed local edges to be preserved in the smoothing of lobules [5,6]. For each pixel, this approach considers a current cubic window of size L= L_x x L_y x L_z, centred on the current voxel. This window is composed of N_x x N_y x N_z sub-windows, of size S_x x S_y x S_z (in voxels). On each sub-window, the average grayscale texture is computed and then the central voxel is set to the value of the median of all means. For algorithmic optimality, when performing the computation in one pixel, the benefit of the previous computations is kept for its neighbour. Thus only the contribution of each new surface within each cubic-window is evaluated, whilst disregarding the contribution of lost surfaces, as is usual in sliding window methods. The algorithmic cost is thus $O(N_xN_yN_zL^3)$ with $N_{x,}$ N_y and N_z corresponding to the image dimensions in the x, y, and z directions. Varying the size of the window, the number of sub-windows and their sizes showed that N_x x N_y x N_z = 3x3x1, S_x x S_y x S_z = 3x3x3, so L = L_x x L_y x L_z = 9x9x3 gave the best results for edge preservations and graylevel smoothing within the lobules. In b2) the image was thresholded for further use in the work-flow. Various threshold values were tested.

c) A simple threshold was used in order to binarize the filtered lobules (Figure 1c).

d) Then the Euclidean distance map to the binarized lobules was computed so as to be able to separate the various entities by a simple thresholding on the distance map (Figure 1d1). This procedure is similar to a mathematical morphology closure but permits elongated throats and bridges to be cut while preserving the shape of the lobules. Step 1d2) is the computation of the gradient of the thresholded lobules using the classical Sobel

method.

e) We proceeded with the thresholding of the distance map, cleaning the smallest islands, labelling the connected components and evaluating each connected component barycentre, to be used as a seed in step f).

f) Seeding step.

g) Finally, we combined the seeding step (f) with identified non-overlapping territories for each lobule and the gradient step (d2) to provide the barriers of the watershed step. Each connected component was associated with a colour-coded representation of the label.

h) A simple thresholding of the original grayscale image (b2) which was then multiplied (pointwise multiplication) by the watershed mask (g), was used to illustrate the quality of the labelling in Figure 1h. In this figure, it can be seen that very small imperfections in the frontier of watershed domains can result in small multi-coloured sets of points at lobule edges. A final correction step to eliminate residual over-segmented watershed regions was performed using a graph merging procedure. Each segmented region was described as the node of a graph, and was connected with other nodes when the watershed regions were neighbours. The edges of the resulting graph were weighted by the number of neighbouring pixels normalized by the average surface of the two lobules in contact ($\frac{S_{contact}^{i,j}}{(S_i+S_j)/2}$for two lobules having labels i and j). The graph-merging procedure consisted of merging two edges when their edge weight was larger than a threshold. This threshold was selected as a quartile of all edge weights. The result is illustrated Figure 1i where the small isolated red watershed region inside the blue region visible in Figure 1h has been merged. Similar ideas of merging areas by a graph representation have already been used in various contexts [7,8]. The final result of the lobule segmentation seems to be improved by this last step, the main interest of which is to eliminate over-connected segmented lobules. The gradient procedure that defines the walls for the watershed might

still have some “holes” separating entities that would not be disconnected otherwise.

In the following table, we provide the list of parameters adjusted for the four segmentations (3 with autofluorescence signal and one with lectin signal):

Work-flow step	Inguinal fat pad n°1	Inguinal fat pad n°2	Inguinal fat pad n°3
SLA mean-filter<br>(x, y, z)	35x35x15	35x35x20	50x35x15
B2 (threshold)	100	90	70
B1 (Kuwahara-like mask)<br>number of windows (x, y ,z)<br>size of window (x, y, z)	3x3x1<br><br>3x3x3	3x3x1<br><br>3x3x3	3x3x1<br><br>3x3x3
C (threshold)	50	120	70
C (fill holes)	400	200	125
E (threshold)	70	100	50
F (size of smallest connected components removed)	2500	2500	1500
I (percentage of merged lobules)	80%	90%	80%

Supplementary table S1: Parameters of the segmentation workflow for the three fat pads from mice maintained at 22°C.

Work-flow step	Inguinal fat pad n°4	Inguinal fat pad n°5
SLA mean-filter<br>(x, y, z)	25x25x15	25x25x15
B2 (threshold)	70	110
B1 (Kuwahara-like mask)<br>number of windows (x, y ,z)<br>size of window (x, y, z)	3x3x1<br><br>3x3x3	3x3x1<br><br>3x3x3
C (threshold)	90	110
C (fill holes)	300	300
E (threshold)	40	115

F (size of smallest connected components removed)	1000	500
I (percentage of merged lobules)	80%	--

Supplementary table S2: Parameters of the segmentation workflow for the two fat pads from mice exposed to 4°C for 48 hours.

Let us finally discuss the limitation of the study associated with the lobule segmentation procedure. It must be pointed out that, since several arbitrary parameters (such as the binary thresholds) were used through the work-flow, the results might be different if other parameters were chosen. Due to the lack of benchmark and/or ground truth for segmented lobules, image analysis validation used an expert's visual segmentation. Another limitation of the method concerns the previously described border artefacts of the watershed, which are generic. Since the segmented structures are complex and heterogeneous, the watershed can over-connect some of them, which will thus not stop at the precise frontier of the "real" lobule. The segmentation of the "core-region", the filtering, and the graph merging were implemented to reduce the spread of the watershed, but some artefacts might still persist, resulting in spurious, hopefully limited, very small subunits.

Robustness of segmentation method:

In this section we investigated the influence of parameter variations in the segmentation results. Focusing on fat pad n°1, we modified the arbitrary parameters at steps C, E and I (used in table S1) for three values (5, 10 and 20% variations) and studied their effects at a given slice in the tissue sample. Once a parameter of a given step was changed, all other parameters were kept identical to study its specific influence. Size of the Kuwahara-like mask and its sub-windows as well as the type quantity computed in each sub-window at step B1 were not investigated on this section because of its computational time on the full 3D image and the many parameters involved. However, its qualitative effects were studied

on small crops of tissue for several sub-windows sizes, sub-window numbers and quantity computed on each sub-window (variance, mean and median). The previously given parameters were seen to provide the best homogenization of gray levels as well as the preservation of contrasts between entities. The size influence of the smallest connected component was not investigated since it is in direct relation with the percentage of merged lobules in step I. Indeed, keeping larger connected components in step F (respectively smaller) will lead to a smaller (respectively larger), percentage of merged lobules in step I. Finally, threshold in B2 wasn't investigated as it is mostly a visualization parameter and its influence would be mainly focussed on merging step I. Considering the image dynamic, the relative differences in terms of normalized contact-surface between pre-segmented entities wouldn't change much from the threshold at step B2.

Figure S1 shows the segmentation obtained for fat pad n°1 with different parameters at depth z = 487.05 µm. Reference (obtained with parameters described in table S1 and illustrated in figure S2A, top panel) is displayed for direct comparison. Colors of segmented units were attributed to the spatially closest units based on reference segmentation. Supplementary table S3 summarizes the parameters used for this study. The results show that the most critical arbitrary parameter is the threshold at step B. With a variation of 5%, the final segmentation keeps the main characteristics of the reference segmentation with a large central pink subunit (Figure S1 b1) but a higher threshold by a 20% variation will lead to over segmented subunits as illustrated in Figure S1 b2. Size of gap filling is quite robust and leads to almost identical segmentations (Figure S1 c1 and c2) as is the threshold on step E (Figure S1 d1 and d2). Most of the segmented subunits are the same as in reference segmentation although one main subunit close to the lymph node in reference segmentation (dark blue color) is now divided in two subunits (Figure S1 d1 and d2). Finally, changing the number of merged subunits (Figure S1 e1 and e2) lead to having two main central subunits instead of the main pink one.

We also quantify for each new 2D crop image, the relative difference with the reference from computing the relative mutual information (relative-MI) [9] as the ratio of the maximum MI, i.e. the MI between the reference image and itself, and the MI between the new segmented image. The results are displayed on supplementary table S3. The higher the relative-MI is, the closer is the image to the reference. As illustrated in Figure S1, the lowest relative-MI value is obtained for 20% threshold variations at step C (Relative-MI = 82%, table S3) as it is the least similar segmented image compared to the reference.

Although the segmentations lead to sometimes slightly different results, it must be kept in mind that we changed only one parameter for each segmentation step and kept the others identical. During the segmentation workflow, if discrepancies appear in one intermediate image or within the final one, backward steps can be applied and new parameters adapted to get a more realistic final segmentation. Such backward steps were not used here which can explain the differences compared to the reference segmentation.

Work-flow step	reference image	C (threshold) +5%	C (threshold) +20%	C (fill holes) +5%	C (fill holes) +20%	E (threshold) +5%	E (threshold) +20%	I (% of merged lobules) +5%	I (% of merged lobules) +20%
C (threshold)	70	74	84	70	70	70	70	70	70
C (fill holes)	125	125	125	132	150	125	125	125	125
E (threshold)	50	50	50	50	50	53	60	50	50
I (percentage of merged lobules)	80%	80%	80%	80%	80%	80%	80%	84%	96%
Relative MI of maximum MI	100%	97%	82%	91%	91%	90%	87%	98%	91%

Supplementary table S3: Consequences of changes in the value of segmentation parameters. Last line reports the relative mutual information (MI) between the reference segmentation image and the new segmentation image obtained with a given set of parameters (Fig. S5). The relative-MI was expressed as the ratio between the maximum-MI obtained with the two images.

Timing of the segmentation method:

Supplementary table S4 provides timing (in seconds) at each step on a 1.5 GB 3D image (fat pad n°3), on a Intel i7 Core 33MHz with 12 processors and 125GB of ram.

Work-flow step	Computational times (in seconds)
B2 (threshold)	110
B1 (Kuwahara-like mask)	18510
C (threshold)	3
C (fill holes)	89
D1 (distance map)	300
D2 (gradient)	174
E (threshold)	3
F (connected components)	33

Supplementary table S4: Computational times of the segmentation of fat pad n°5 corresponding to an image size of 1.5GB.

Additional discussion about the State of the art

Image segmentation is a long standing topic in image analysis, of particular relevance to biomedical imaging communities. The workflow proposed in this article is original as it has been dedicated to the specific task at end but follows conventional pipelines. Deep-learning methods disrupt the field of image segmentation because they permit robust, performant, and fully automated workflows when possible ([10-13]). Deep-learning methods outperform traditional approaches when it is possible to train the algorithms on huge databases onto which ground truth segmentation is available. Nevertheless these supervised approaches are often a distressing step on large 3D images. Manual segmentation for building training data-set remains a big challenge for specific and dedicated task. When the segmentation concerns cancer tumors detection, it is easy to find among the radiologists community the required man power and motivation to build

such ground-truth data-set, because this task is indeed a crucial daily-based, intensely sensitive issue for a huge medical community. Regarding the topic of interest here, there is no chance of building the required training data-base of 3D fat-pad lobules. The building of a training data base is not only a costly and intensive issue, but also requires a huge amount of 3D images not possible to provide in our case. Hence for the issue at end in this very specific set of images, we did not found possible to use a deep-learning approach for 3D-lobules segmentation.

References

1 Barreau, C. *et al.* Regionalization of browning revealed by whole subcutaneous adipose tissue imaging. *Obesity* **24**, 1081-1089, doi:10.1002/oby.21455 (2016).

2 Preibisch, S., Saalfeld, S. & Tomancak, P. Globally optimal stitching of tiled 3D microscopic image acquisitions. *Bioinformatics* **25**, 1463-1465, doi:10.1093/bioinformatics/btp184 (2009).

3 Schindelin, J. *et al.* Fiji: an open-source platform for biological-image analysis. *Nature methods* **9**, 676-682, doi:10.1038/nmeth.2019 (2012).

4 Jiang, M. Chan Tony F, Shen Jianhong (Jackie): Image Processing and Analysis: Variational, PDE, Wavelet, and Stochastic Methods. *Biomedical engineering online* **5**, 38, doi:10.1186/1475-925x-5-38 (2006).

5 Kuwahara, M., Hachimura, K., Eiho, S. & Kinoshita, M. *Processing of RI-Angiocardiographic Images*. (1976).

6 Schulze, M. A. & Pearce, J. A. in *Proceedings of the 1994 IEEE International Conference on Image Processing (ICIP-94).* 530-534.

7 Vadiveloo, M., Abdullah, R. & Rajeswari, M. in *Seventh International Conference on Graphic and Image Processing.* 9 (SPIE).

8 Blaffert, T., Renisch, S., Schadewaldt, N., Schulz, H. & Wiemker, R. in *SPIE Medical Imaging.* 9 (SPIE).

9 Cover, T. M. & Thomas, J. A. *Elements of Information Theory (Wiley Series in Telecommunications and Signal Processing).* (Wiley-Interscience, 2006).

10 Ajmal, H. *et al. Convolutional neural network based image segmentation: a review*. Vol. 10649 SID (SPIE, 2018).

11 Akkus, Z., Galimzianova, A., Hoogi, A., Rubin, D. L. & Erickson, B. J. Deep Learning for Brain MRI Segmentation: State of the Art and Future Directions. *J Digit Imaging* **30**, 449-459, doi:10.1007/s10278-017-9983-4 (2017).

12 Zhao, B., Feng, J., Wu, X. & Yan, S. A survey on deep learning-based fine-grained object classification and semantic segmentation. *Int. J. Autom. Comput.* **14**, 119-135, doi:10.1007/s11633-017-1053-3 (2017).

13 Xu, Y. *et al.* Large scale tissue histopathology image classification, segmentation, and visualization via deep convolutional activation features. *BMC Bioinformatics* **18**, 281, doi:10.1186/s12859-017-1685-x (2017).

14 Risser, L., Plouraboué, F. & Descombes, X. Gap Filling of 3-D Microvascular Networks by Tensor Voting. *IEEE transactions on medical imaging* **27**, 674-687, doi:10.1109/TMI.2007.913248 (2008).

Supplementary figures:

Supplementary Movies 1 and 2: Different morphologies of two segmented polylobular subunits of the fat pad. Smoothed polylobular subunits are obtained by applying a mean filter followed by several successive dilatation and erosion steps. Movie 1 illustrates the elongated dark green subunit located below and to the right of the lymph node on Figure 4A. Movie 2 illustrates the cyan subunit connected to 7 other subunits (two blue, two pink, one violet, one dark green and one light green) in Figure S1B.

Supplementary Movie 3: Preferential alignment of segmented subunits. This movie shows a skeleton superimposed on smoothed subunit shown in Movie 1. A. The skeleton was computed using the method reported by Risser et al.[14] and then simplified by removing smallest segments.

Supplementary figure S1: The segmentation procedure gives similar results when individual parameters are varied by 5% (left column) and 20% (right column). (A) The reference segmentation of fat pad n°3 at a given slice (z = 234.36 µm) obtained by using parameters described in supplementary table S1. Segmentation results for varying threshold of step C (b1 and b2), size of holes filled of step C (c1 and c2), threshold of step E (d1 and d2) and percentage of merged units of step I (e1 and e2).

Supplementary figure S2: The segmentation procedure gives similar results with two different signals, i.e. autofluorescence (a) and lectin signal (b) in one tissue sample. A) The images have been aligned around the lymph node, which is the rounded purple shape, the correspondence of which between the two images is sketched with the white dotted line. To illustrate the similarities between subunit segmentations, we have

used the same colour label on subunits that are qualitatively similar. B) Quantification of the volumes of the different segmented subunits identified by the acquisition of the autofluorescence (left panel) and lectin (right panel) signals. Histograms represent the number of subunits displaying equivalent volumes (from the smallest, i.e. <2mm^3 to the largest, i.e. >5 mm^3) identified by the two segmentation procedures.

Supplementary figure S3: 2D Graph representation of the subunit locations, connections and sizes in the segmented zone for samples n°3 (A) and n°1 (B). The (x,y) coordinates indicate the position on a slice. The positions of the nodes of the graph, i.e. the centre of each circle, are those of the barycenters of the subunits. The radius of the circle is proportional to the volume of the subunits. The links between two nodes are computed as the ratio of the contact surface between two subunits to their mean total surface. Thus, the larger the link is, the stronger is the connection between two subunits. The grey and red dotted circles in each figure illustrate the two clusters of well-connected subunits. LN, Lymph node.

Supplementary figure S4: The two clusters of subunits present distinct biological characteristics that could be predictive of browning abilities. Immunostaining of TOM20 (red, Figure A) and tyrosine hydroxylase (green, Figure B) performed on areas 1, 2 and 3 of inguinal fat pad sections of C57Bl6 mice exposed to 22 °C. LN, lymph node. Scale bar corresponds to 100 µm (A) and 50 µm (B). C) Quantification of sympathetic nerve density on areas 1, 2 and 3. D) Proxy of vascular density on 3D regions of interests of the lectin-labelled sample n°3. *$p < 0.05$, **$p < 0.01$ and ***$p < 0.001$ comparison between areas.

Supplementary figure S5: Cold exposure does not change architecture of inguinal fat pad. A) Relative volume of segmented subunits. B) 2D Graph representation of the subunit locations, connections and sizes. The grey and red dotted circles in each figure illustrate the two clusters of well-connected subunits. LN, Lymph node.

Supplementary Figure S1

Dichamp J. *et al.*

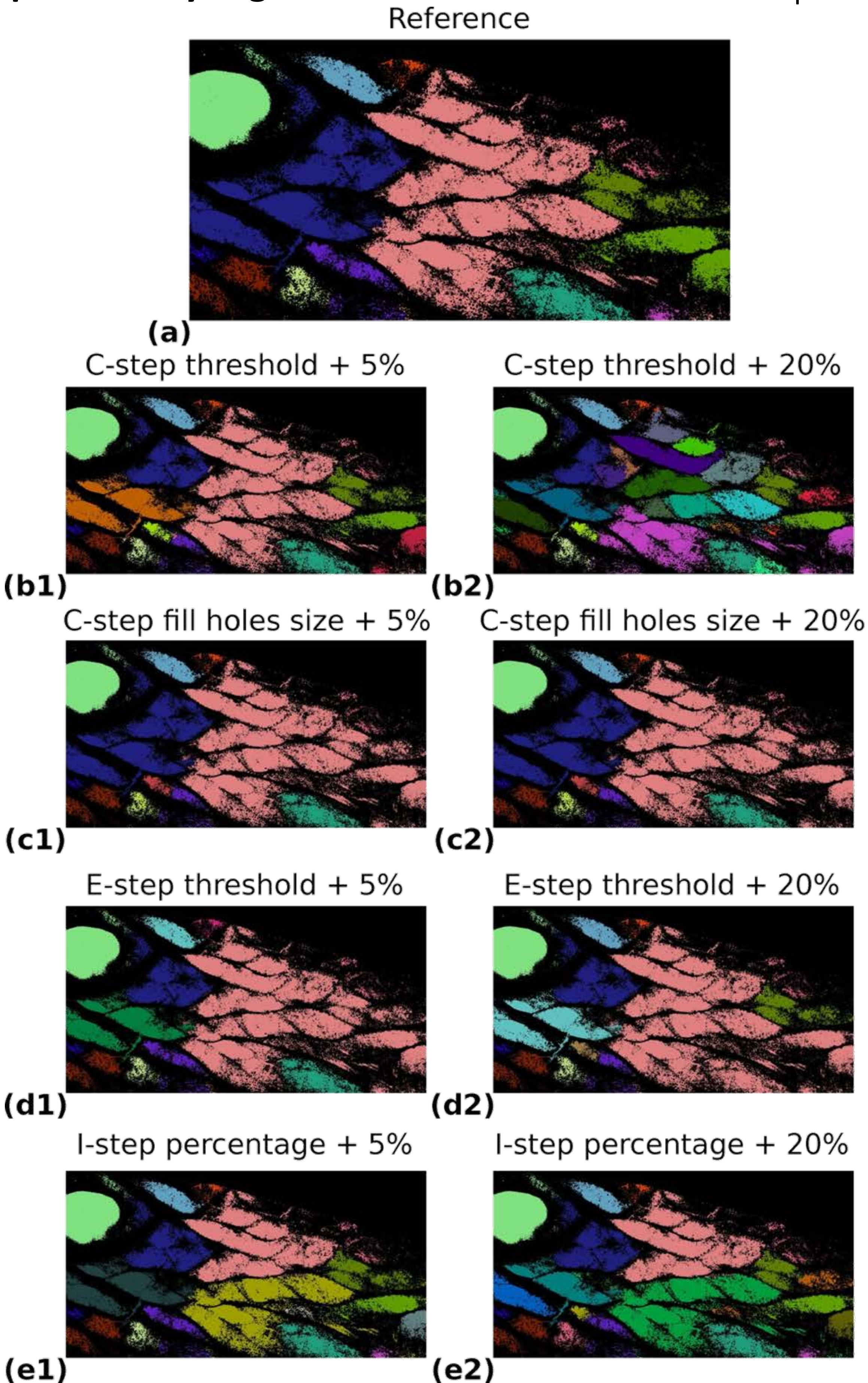

Supplementary Figure S2

Dichamp J. *et al.*

A.

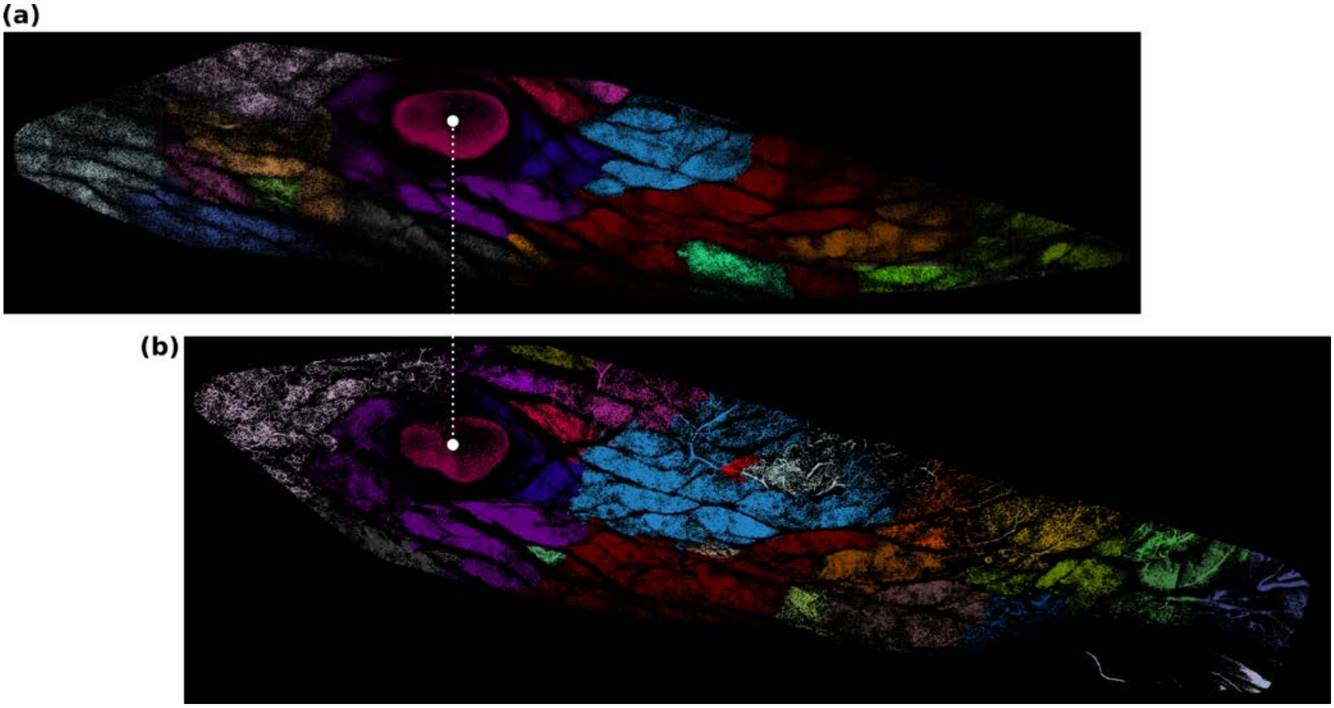

B.

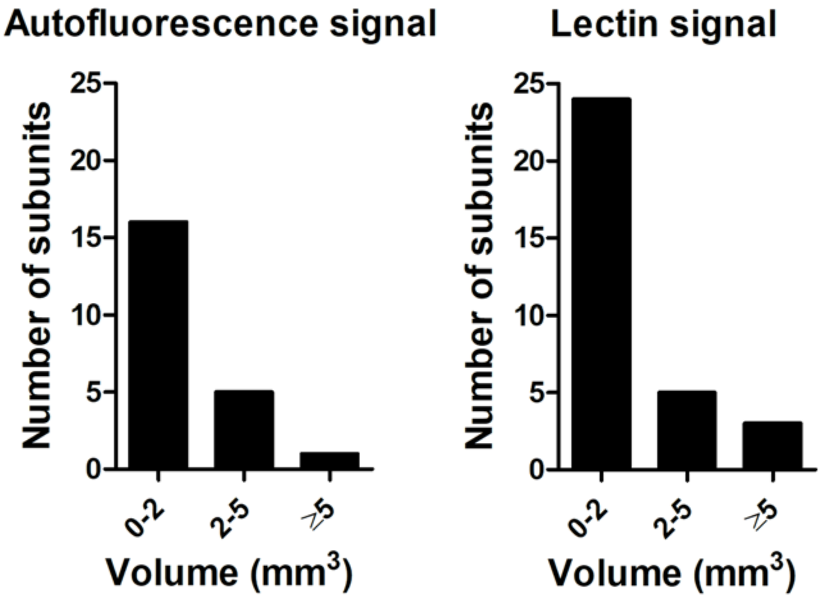

Supplementary Figure S3

Dichamp J. *et al.*

A.

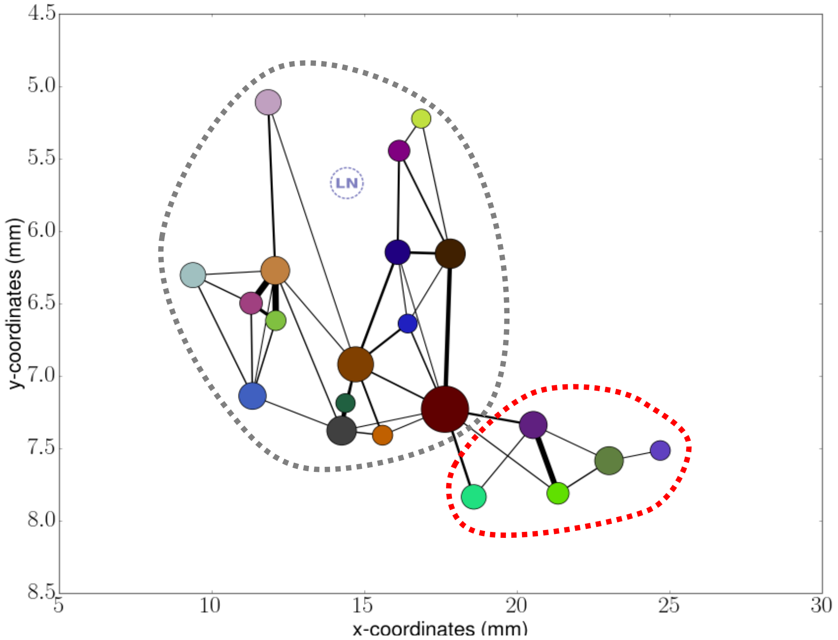

B.

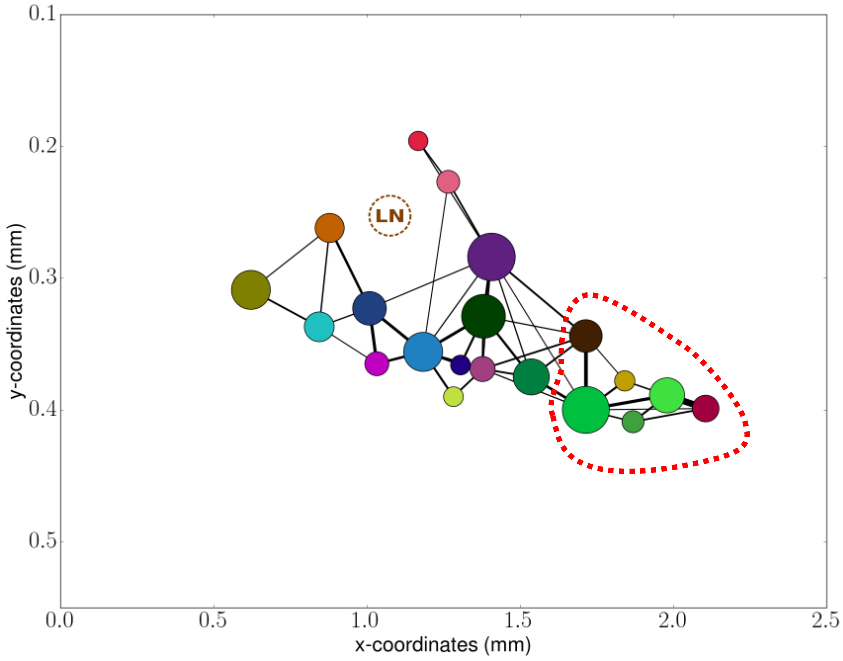

Supplementary Figure S4

Dichamp J. *et al.*

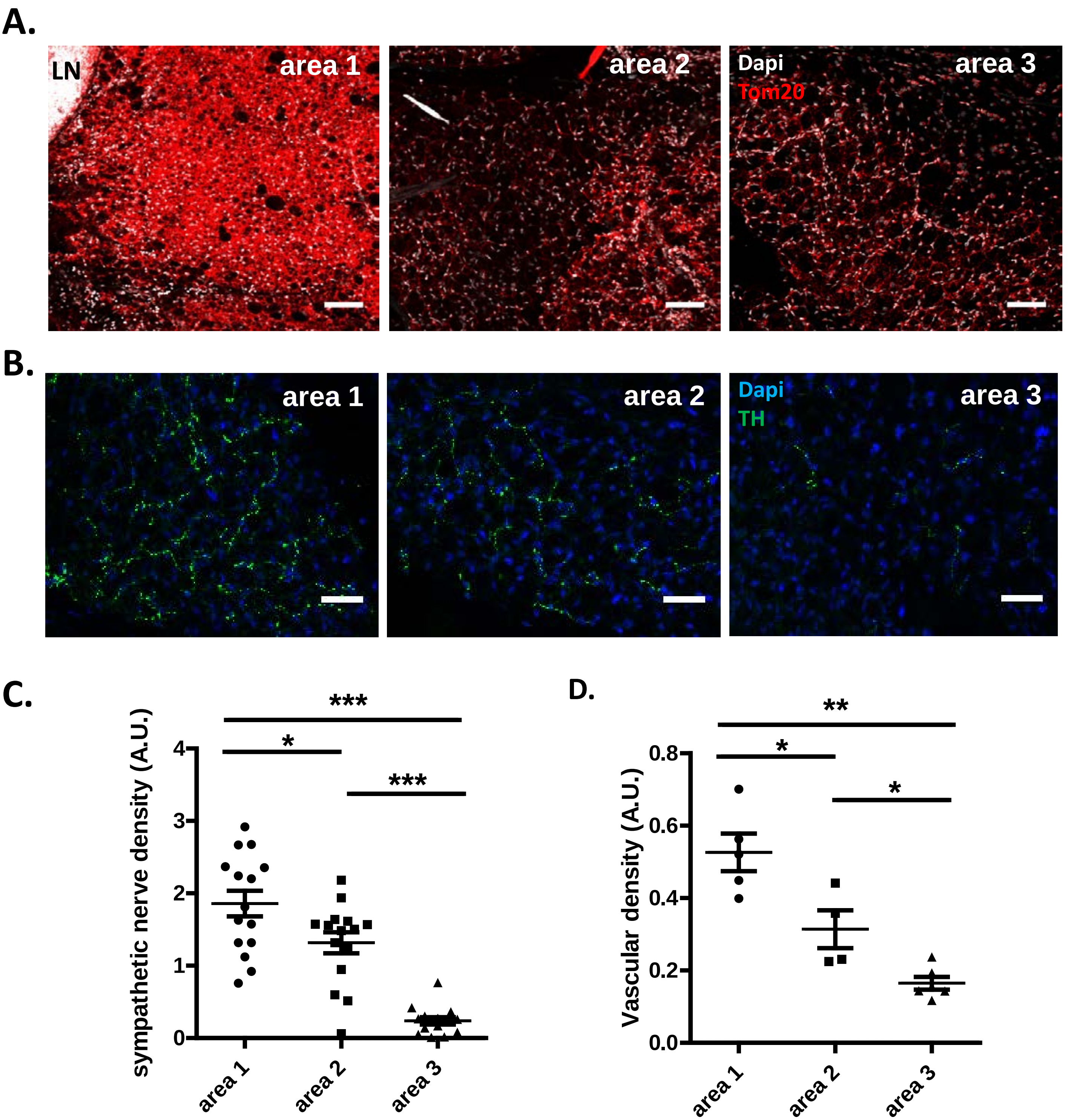

Supplementary Figure S5

Dichamp J. *et al.*

A.

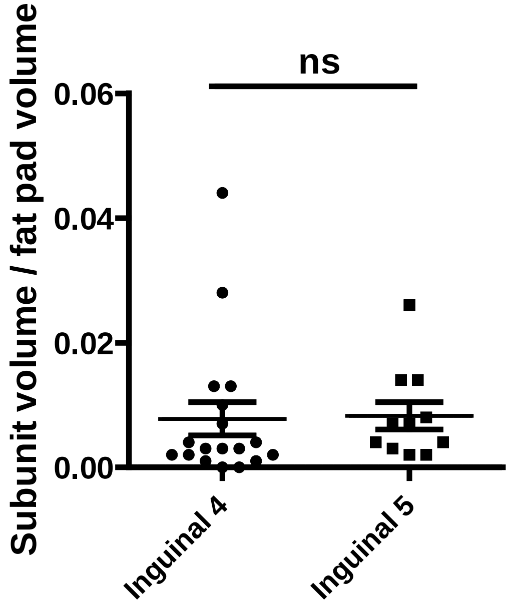

B.

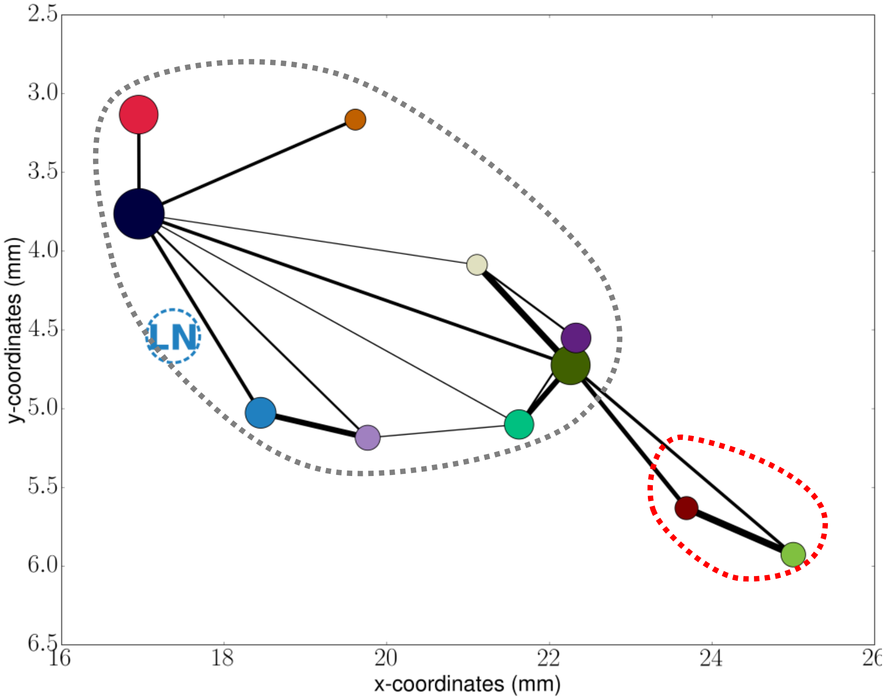

Supplement: Supplementary file 1 — Supplementary Information [file 41598_2019_43130_MOESM1_ESM.pdf]
